# Unpacking Tourette Syndrome in Children: Insights into Prevalence and Comorbidities from NSCH Data

**DOI:** 10.3390/jcm14051485

**Published:** 2025-02-23

**Authors:** Sasidhar Gunturu, Mahdieh Saeidi, Omar Alzein, Kamyar Jafari, Mona Salehi, Sanobar Jaka

**Affiliations:** 1 Department of Psychiatry, Bronx Care Health System, Bronx, NY 10457, USA; 2 Department of Psychiatry, Icahn School of Medicine at Mount Sinai, New York, NY 10029, USA; 3 Department of Psychiatry, School of Medicine, University of Minnesota, Minneapolis, MN 55455, USA; 4 Department of Psychiatry and Behavioral Sciences, Nassau University of Medical Center, East Meadow, NY 11554, USA

**Keywords:** Tourette syndrome, prevalence, comorbidities, children, national survey of children’s health, neurodevelopmental disorders

## Abstract

**Background:** Tourette syndrome is a neurodevelopmental disorder characterized by multiple motor and vocal tics. Although Tourette syndrome is known to have various comorbidities, comprehensive data on its prevalence and associated conditions in a large, diverse population are limited. This study aimed to examine the prevalence of Tourette syndrome and its comorbidities in children aged 3 to 17 years using data from the 2021 National Survey of Children’s Health (NSCH). **Methods:** Data from 79,236 children aged 3–17 years were analyzed. The prevalence of Tourette syndrome was assessed, and its association with socio-demographic factors and comorbid conditions, including prematurity and low birth weight, was examined using univariate and multivariate logistic regression models. **Results:** The prevalence of Tourette syndrome was 0.3% among children aged 3–17 years, with higher rates in males (74%) and adolescents aged 11–17 years (74%). Prematurity and low birth weight were associated with higher rates of Tourette syndrome and its comorbidities. Neurodevelopmental conditions such as ADHD (49% in Tourette syndrome vs. 10.2% in non-Tourette syndrome), autism spectrum disorder (21% vs. 3.2%), and learning disabilities were significantly more prevalent among children with Tourette syndrome. Similarly, psychiatric disorders such as anxiety (60% vs. 11.3%) and depression (25% vs. 5%) were more common in the Tourette syndrome group. Immune-based conditions, including asthma and allergies, and physical health conditions such as diabetes and vision or hearing problems, were also significantly associated with TS. **Conclusions:** The study highlights the significant burden of comorbidities in children with Tourette syndrome, emphasizing the need for early diagnosis and comprehensive management strategies to address the multifaceted challenges faced by these children.

## 1. Introduction

Tourette syndrome (TS) is a neurodevelopmental disorder first described by Georges Gilles de la Tourette in 1885 [1]. Initially, Tourette proposed hereditary degeneration as its cause, a theory that was later refuted through advancements in scientific understanding. Early 20th-century research linked tics to conditions like chorea, rheumatic fever, or psychological conflicts [2,3,4,5]. However, in the 1960s, the discovery of haloperidol’s effectiveness in treating tics shifted the understanding from psychosexual theories to neurological causes [6]. By the 1970s, the formation of the Tourette Syndrome Association (TSA) contributed to the current view of TS as a disorder with genetic and neurological origins, involving dysfunctions in dopamine signaling and the basal ganglia [7].

TS is characterized by repetitive, involuntary movements and vocalizations known as tics. These tics can vary significantly in severity, frequency, and complexity across individuals. Motor tics may involve movements such as eye blinking or shoulder shrugging, while vocal tics can range from simple sounds like throat clearing to more complex vocalizations [8]. Although the precise cause of TS remains unknown, it is widely accepted that a combination of genetic and environmental factors, along with abnormalities in neurotransmitters like dopamine, play a central role in its pathophysiology [9].

The study of TS in children is critical because the disorder typically begins in early childhood, usually between the ages of 4 and 6, with peak severity occurring around 10 to 12 years of age. Early diagnosis and intervention are essential for managing symptoms and minimizing the impact on the child’s quality of life [10,11]. Children with TS often experience a reduced quality of life due to the disruptive nature of their tics and the effort required to suppress them, which can be further complicated by comorbid conditions such as attention-deficit hyperactivity disorder (ADHD) and anxiety. These comorbidities exacerbate social isolation, poor academic performance, and lower self-esteem, significantly increasing the disease burden [11,12].

Low birth weight and prematurity are among the environmental factors increasingly recognized as relevant in the pathogenesis of TS. Research indicates that prenatal and perinatal adversities, including low birth weight and prematurity, may influence the onset and severity of TS symptoms. Although the precise mechanisms remain unclear, it is hypothesized that these factors may contribute to early brain injury or disruptions in the dopaminergic system, a pathway critical to the neurobiology of TS. Studies have shown an association between prematurity and an increased risk of tic severity, while low birth weight has been linked to an elevated risk of TS comorbidities such as ADHD and obsessive-compulsive disorder [13,14].

Furthermore, TS can place a substantial strain on family dynamics. Families often face heightened stress, challenges in managing the child’s care, and disruptions to daily routines. These factors collectively emphasize the importance of studying TS in children not only to improve individual outcomes but also to alleviate the broader impact on family well-being [15].

TS is clinically defined by the presence of multiple motor tics and at least one vocal tic, lasting for more than a year with an onset before the age of 18. These tics are often preceded by a premonitory urge, which can be difficult to resist and may need to be repeated until they feel “just right”, providing temporary relief. The frequency and intensity of tics can fluctuate, often worsening during periods of stress or excitement, and improving during focused activities. Importantly, the condition is not caused by substance use or other medical disorders [16,17].

The prevalence of TS in North America is estimated to range between 3 and to 9 per 1000 school-age children, with a male-to-female ratio ranging from 2:1 to 4:1. Globally, TS is more common in boys, and research suggests that a combination of genetic, neurological, and inflammatory factors contributes to its development. Genetic pathways, immune responses, and neural circuitry are all implicated in the disorder’s etiology, and ongoing research continues to investigate these connections to improve treatment options [16,18,19].

Despite extensive research on TS, there is a critical need for large-scale, population-based studies to more comprehensively understand its prevalence and comorbidities in children. Previous studies have been limited by small sample sizes and geographic constraints. The National Survey of Children’s Health (NSCH) dataset offers a valuable opportunity to overcome these limitations, providing data that can significantly inform clinical practice and public health policy. While prior analyses of the NSCH have offered insights into TS, the most recent comprehensive analysis dates to 2012–2014 [20].

This study aims to address several critical gaps in the understanding of TS. By analyzing the 2021 NSCH dataset, this research seeks to update the prevalence and comorbidity data to provide actionable insights for policymaking and resource allocation. Understanding the comorbid conditions associated with TS will contribute to a better understanding of its pathophysiology and may lead to more effective, evidence-based treatment plans. This study addresses the lack of updated data by analyzing the 2021 NSCH, thus offering a refreshed perspective on TS prevalence and comorbidities in the pediatric population.

## 2. Materials and Methods

### 2.1. Data Source and Study Design

This study utilized data from the 2021 National Survey of Children’s Health (NSCH), a nationally representative cross-sectional survey. The NSCH is conducted annually by the US Census Bureau and funded by the Health Resources and Services Administration’s Maternal and Child Health Bureau [13]. The survey gathers comprehensive information on the health and well-being of noninstitutionalized children aged 0–17 years across the United States [21].

### 2.2. Study Population

The analysis included 79,236 children aged 3 to 17 years. The NSCH employed a multistage sampling design, selecting addresses from the Census Bureau’s Master Address File (MAF). Households were selected randomly, and one child per household was chosen to complete the detailed topical questionnaire. Data collection covered all 50 states and the District of Columbia [22]. The response rates were 40.3% in 2021. Sampling weights were applied to account for nonresponse bias and to ensure the data were representative of the national population [23].

### 2.3. Data Collection Procedure

The NSCH utilized a two-phase data collection methodology. Initially, a household screener determined the presence of children and their basic demographic characteristics. Following this, a detailed questionnaire was administered to parents or caregivers of the selected child. The survey included both web-based and mail-in options to accommodate different respondent preferences and to improve response rates. Strategies such as clear question phrasing, multiple response mode options, and incentives were employed to enhance participation [21].

The NSCH 2021 data were subjected to a comprehensive weighting process to correct for nonresponse and ensure representativeness. This process included post-stratification adjustments and a technique known as raking. Raking adjusted the case weights repeatedly across various dimensions, including state-by-household poverty ratio, respondent education level, race/ethnicity of the selected children, age group, and national distributions of children’s race/ethnicity and sex. This process was repeated for up to 100 cycles or until the weights accurately reflected population totals. Additional information on the selection and sampling methodology is available on the Data Resource Center (DRC) website at https://www.childhealthdata.org/learn-about-the-nsch/NSCH (accessed on 20 February 2025).

The study also included analyses of prematurity and low birth weight, as defined by parental responses to specific survey questions. In the survey, prematurity was defined as being born more than three weeks before the due date, and low birth weight as a birth weight of less than 2500 g. These variables were examined as independent predictors of TS and related comorbidities. Subgroup analyses were conducted to assess the socio-demographic and clinical characteristics of children with TS who were born prematurely or with low birth weight.

### 2.4. Confidentiality

Participation in the NSCH was voluntary, and all data were collected in compliance with federal confidentiality laws, specifically 13 U.S.C. Section 9 and Section 23(c). The data were de-identified and subjected to rigorous disclosure review processes before being made publicly available, ensuring respondent privacy and data security [23].

### 2.5. Measures

#### 2.5.1. Independent Variables

Socio-demographic variables considered in this analysis included age, sex, race/ethnicity, family income relative to the federal poverty level (FPL), and the highest education level of adults in the household. The primary outcome variable was the presence or absence of TS, as reported by parents or caregivers. They were asked “*Has a doctor or other healthcare provider ever told you that this child has Tourette Syndrome*?” (Supplementary S1). Prematurity and low birth weight were also included as key variables to evaluate their potential associations with TS and its comorbid conditions.

#### 2.5.2. Comorbid Conditions

The analysis also included various comorbid conditions. Included among the neurodevelopmental and behavioral disorders are ADHD, autism spectrum disorders (ASDs), developmental delay, Down syndrome, cerebral palsy, learning disability, and behavioral and conduct problems. Included psychiatric and neuropsychiatric disorders were depression, anxiety problems, headaches, and seizures. Also considered were immune-related conditions such as allergies and asthma, and the physical health conditions included in the analysis were diabetes, heart disorders, genetic disorders, vision problems, and hearing problems.

#### 2.5.3. Statistical Analysis

Statistical analyses were conducted using Stata version 18.0 [24]. Continuous variables were summarized as mean ± standard deviation, while categorical variables were presented as frequencies (percentages). Initial comparisons between children with and without TS were performed using *t*-tests for continuous variables and chi-square tests for categorical variables. Univariate and multivariate logistic regression models were employed to examine associations between socio-demographic factors, comorbid conditions, and TS. Univariate models independently assessed each covariate’s association with TS, while multivariate models adjusted for all covariates. Adjusted odds ratios (ORs) and 95% confidence intervals (CIs) were reported to indicate the likelihood of TS associated with each covariate after controlling for other factors.

## 3. Results

This study analyzed 79,236 participants, of whom 208 (0.26%) had Tourette syndrome (TS). Participants with TS were older, with a mean age of 12.7 years compared to 10.0 years among those without TS (Table 1). Adolescents (11–17 years) constituted most of the TS group (74%), a figure significantly higher than their representation in the overall population (48%). Males accounted for 74% of individuals with TS, highlighting a marked sex disparity.

### 3.1. Socio-Demographic Predictors of TS

Multivariate analysis identified adolescence as a strong predictor of TS, with adolescents having 7.4 times higher adjusted odds (95% CI: 4.0–14.1, *p* < 0.001) compared to preschool-aged children (3–5 years) (Table 2). Females were found significantly less likely to have TS compared to males (adjusted OR: 0.4, 95% CI: 0.3–0.5, *p* < 0.001). Moreover, socioeconomic factors were determined to influence TS risk, as participants from households with incomes between 200% and 399% of the federal poverty level (FPL) had lower adjusted odds (OR: 0.6, 95% CI: 0.4–0.9, *p* = 0.020) compared to those from households with incomes below 100% FPL.

### 3.2. Comorbid Conditions

Comorbid conditions were significantly more prevalent among individuals with TS. Overall, 79.8% of individuals with TS had at least one comorbid condition (Table 3). The most common comorbid condition was attention-deficit hyperactivity disorder (ADHD), affecting 49% of individuals with TS compared to 10.2% of those without TS (*p* < 0.001). Anxiety disorders were present in 60% of the TS group, significantly higher than the 11.3% observed in the non-TS population (*p* < 0.001). Depression (25% vs. 5%, *p* < 0.001), autism spectrum disorder (ASD; 21% vs. 3.2%, *p* < 0.001), and developmental delays (23% vs. 6%, *p* < 0.001) were also notably higher among TS participants. Learning disabilities affected 28% of individuals with TS, compared to 7.5% of those without TS (*p* < 0.001).

### 3.3. Subgroup Analysis: Prematurity and Low Birth Weight

Subgroup analyses revealed distinct socio-demographic and clinical profiles for children with TS born prematurely (<37 weeks) or with low birth weight (<2500 g). Among the 36 TS participants born prematurely, the majority were adolescents (75%) and male (69.4%), consistent with the overall TS population. Similarly, the 27 individuals with TS and low birth weight were predominantly adolescents (70.3%) and male (70.4%) (Table 1). However, Hispanic children were overrepresented in the TS + low birth weight subgroup (22.2%) compared to the overall TS group (14%).

Children with TS born prematurely or with low birth weight had higher rates of comorbid conditions. Notably, ASD was significantly more prevalent in the TS + prematurity (32.3%) and TS + low birth weight (40%) subgroups compared to the overall TS group (21%) (Table 3). Asthma and allergies were also more common in these subgroups, with asthma affecting 37.1% of the TS + prematurity subgroup compared to 15.5% of the overall TS group.

## 4. Discussion

The present study identified a prevalence rate of TS of approximately 0.3% in children aged 3 to 17 years, which is consistent with previous reports that have estimated TS prevalence to range from 0.3% to 0.8% in school-aged children. This study confirms prior findings that TS is significantly more prevalent in males and adolescents aged 11–17 years [25]. Furthermore, we found several comorbid conditions with TS, including neurodevelopmental disorders, psychiatric disorders, immune-related conditions, and other physical health issues (such as diabetes, heart disorders, cerebral palsy, and sensory impairments like vision and hearing problems).

The higher prevalence of TS among males is well documented in the literature and may be explained by underlying neurodevelopmental and hormonal differences [11,16,26]. Boys with TS often exhibit structural brain variations, such as thinner frontal cortical regions and reduced corpus callosum volume, which might contribute to the severity of their tics [27,28,29,30]. Additionally, testosterone-driven changes in dopamine receptor density and shorter ocular fixation durations have been linked to the greater occurrence of specific tics in males [31,32]. In contrast, older females often experience an increase in tic complexity, potentially due to enhanced brain connectivity between subcortical structures and cortical areas [33].

Regarding race and ethnicity, White children exhibited higher rates of TS, while Hispanic, Black, and Asian children were under-represented, aligning with the hypothesis that diagnostic disparities may exist. These trends highlight the need for culturally sensitive diagnostic practices and equitable access to healthcare services across diverse populations [34,35]. Contrary to expectations, socioeconomic factors such as family income and parental education levels did not significantly differ between children with and without TS, suggesting that genetic and environmental factors may play a more substantial role than socioeconomic conditions in the development of TS [13].

The study revealed that adolescents aged 11–17 years represented the largest group affected by TS, which aligns with the typical onset and peak severity of tics during this developmental stage. Delays in TS diagnosis, often influenced by the nature of the first presenting tics (e.g., motor versus vocal tics), may lead to prolonged diagnostic lag [36,37]. Additionally, the presence of comorbid conditions like obsessive–compulsive disorder (OCD), which is associated with more severe tic presentations, generally tends to reduce the diagnostic delay. In contrast, the absence of such comorbidities can lead to a longer diagnostic lag, as the tics may be less severe and not immediately recognized as part of TS. The variability in diagnosis is further compounded by the multifaceted nature of TS, where symptoms are often masked by other conditions like ADHD and by inconsistencies in diagnostic criteria across different editions of the Diagnostic and Statistical Manual of Mental Disorders (DSM) and World Health Organization (WHO) guidelines [34]. As children age, their tics typically become more prominent and easier to recognize. Furthermore, older children are more frequently screened in school settings, contributing to higher diagnosis rates in this age group [38,39].

### 4.1. Comorbid Neurodevelopmental and Behavioral Disorders

Our analysis revealed significant comorbidities between TS and neurodevelopmental and behavioral disorders. ADHD was found to be notably more prevalent in participants with TS (49%) compared to those without TS (10.2%) (*p* < 0.001). This aligns with Sambrani et al., who found ADHD in 44.9% of patients with TS and noted increased tic severity in TS patients with ADHD, and these individuals often have a lower ability to suppress tics [18]. Adolescents with TS face unique challenges in their learning environments, often due to the visible nature of tics, which can lead to bullying, social isolation, and misunderstanding from peers and educators. These students may struggle with concentration, particularly when coping with the added anxiety of suppressing tics. Co-occurring conditions like ADHD and OCD further exacerbate difficulties in focusing and engaging with schoolwork. The stigma surrounding TS often results in these adolescents being unfairly disciplined, underscoring the need for informed educational interventions [40,41].

The comorbidity between TS and ADHD likely stems from genetic and neurobiological factors. Although specific genes remain unidentified, research by Tian et al. explored the correlation of gene expression between TS patients and the two ADHD subtypes: hyperactivity/impulsivity (HI) and inattention (IA). For the HI subtype, genes such as catechol-O-methyltransferase (COMT), dopamine receptor D2 (DRD2), monoamine oxidase A (MAOA), and solute carrier family 6 member 4 (SLC6A4) showed positive and negative correlations. For the IA subtype, genes previously linked to ADHD, including myelin-associated oligodendrocyte basic protein (MOBP), dopamine receptor D1 (DRD1), and fatty acid desaturase 2 (FASD2), were found to be associated [42,43,44]. Epigenetic factors, including prenatal exposure to alcohol and tobacco, low birth weight, and psychosocial stress, also contribute to this comorbidity [45]. Neuroimaging studies show abnormalities in shared brain regions like the prefrontal cortex and basal ganglia, contributing to both motor control issues in TS and attention deficits in ADHD. Both disorders are linked to dopamine and glutamate imbalances, affecting the cortico-striato-thalamo-cortical circuits [45,46,47,48]. ASD was more prevalent in participants with TS (21%) than in those without TS (3.2%) [*p* < 0.001]. The high rate of comorbid ASD observed in our TS samples aligns with recent findings suggesting a significant overlap between these neurodevelopmental disorders. Research highlights the role of Ash1l, a gene highly expressed in the brain, which correlates with the neuropathology of TS, ASD, and intellectual disability. Ash1l mutations, although rare, have been linked to shared epigenetic factors and overlapping neuropathological mechanisms in these disorders, particularly within the cortico-striatal-thalamo-cortical circuits. Animal studies have further demonstrated the significance of Ash1l in neurodevelopment, providing insights that may lead to therapeutic advances. Reports indicate that ASD prevalence in individuals with TS can reach 20%, especially among those with high-functioning autism. This overlap complicates diagnosis and treatment, emphasizing the need for careful screening and a nuanced approach that addresses both conditions concurrently [49,50,51].

Participants with TS had significantly higher rates of developmental delay (23%) compared to those without TS (6%) (*p* < 0.001). Learning disability was also more prevalent in the TS group (28%) versus the non-TS group (7.5%) (*p* < 0.001). This aligns with findings by Burd et al., who highlighted the potential for shared genetic factors contributing to the comorbidity between TS and learning disabilities [52]. In 1986, Gabor Barabas et al. reported an association between Down syndrome (DS) and TS, suggesting that neurotransmitter abnormalities in DS may predispose individuals to tics due to basal ganglia dysfunction [53]. Our study also found a higher prevalence of DS in TS patients (1.4%) compared to non-TS (0.2%) (*p* < 0.001). Additionally, our study found a higher prevalence of disruptive behavior disorders, such as conduct disorder (CD), in TS participants (36%) compared to non-TS participants (8%) (*p* < 0.001). This may be linked to shared neurobiological and genetic factors, as supported who identified a genetic link between TS and CD, along with attention deficit disorder, depression, and compulsive behaviors [54]. Ahmad Ghanizadeh et al. also observed a high co-occurrence of disruptive disorders, including ADHD and oppositional defiant disorder (ODD), in children with TS [55]. These findings highlight the need for comprehensive management strategies that address both tics and associated behavioral issues to improve patient outcomes.

### 4.2. Psychiatric and Neuropsychiatric Disorders

Depression is significantly more common in individuals with TS (25%) compared to those without TS (5%) (*p* < 0.001). This increase is likely due to the chronic nature of TS, which often leads to social isolation, stress, and low self-esteem, as well as frequently coexisting with other psychiatric disorders like ADHD and OCD. Both TS and depression share neurotransmitter imbalances, particularly involving dopamine and serotonin. Additionally, neuroleptic medications used to manage TS can mimic or exacerbate depressive symptoms, contributing to emotional blunting, anhedonia and fatigue [56,57].

Anxiety is a common comorbidity in individuals with TS, significantly impacting their quality of life. In our study, 60% of participants with TS reported anxiety, compared to 11.3% of those without TS (*p* < 0.001), highlighting the strong link between TS and anxiety. A qualitative study of youth with TS found that anxiety about tics often worsens tic severity, creating a cycle of increased distress. These findings underscore the need to address anxiety in the clinical management of TS to improve overall outcomes [58,59].

Headaches were more common in the TS group (14.4%) versus the non-TS group (4%) (*p* < 0.001), potentially due to serotonin abnormalities that affect both mood regulation and vascular control, contributing to migraines. Ghosh et al. found similar results, with migraines and tension headaches significantly more common in children with TS [60,61].

Seizure disorders were also more prevalent in TS participants (6%) compared to non-TS (0.6%) (*p* < 0.001), consistent with the population-based case-control study conducted by Wong et al. which showed an increased seizure risk in children with TS. Shared dysfunctions in cortico-striato-thalamo-cortical (CSTC) circuits and the autonomic nervous system may explain this heightened risk [62].

### 4.3. Immune-Related Conditions

The high comorbidity of immune-based disorders, such as allergies (37%) and asthma (15.5%), with TS may be due to neuroimmune interactions underlying TS pathophysiology (*p* < 0.001). Neuroinflammation and immune dysregulation are increasingly recognized in TS, with studies suggesting that immune responses influence neurological function. The cortico-striato-thalamo-cortical circuitry, crucial for TS symptoms, interacts with neurotransmitter systems that modulate immune responses, which could lead to a heightened inflammatory state [63,64].

Elevated levels of stress-related immune markers like corticotropin-releasing factor (CRF) and abnormal hypothalamic-pituitary-adrenal (HPA) axis function in TS further link immune dysregulation with TS symptoms [64]. Neurotransmitters like norepinephrine and serotonin, involved in immune regulation and TS pathophysiology, may link TS with immune-based comorbidities. Norepinephrine regulates glucocorticoid secretion, influencing stress responses and immune function. Dysregulation in this neurotransmitter system could contribute to TS symptoms and increased susceptibility to immune-based disorders [64].

Glutamate, the primary excitatory neurotransmitter, plays a key role in TS by affecting specific brain circuits, particularly the CSTC circuits, leading to hyperexcitability and tics. Glutamate dysregulation may also impact neuroinflammatory processes and immune responses in the central nervous system [65,66]. Shared genetic variants affecting both glutamates signaling, and immune regulation could underlie the co-occurrence of TS and immune-related conditions. Additionally, environmental factors like early-life exposure to allergens or infections may trigger immune responses that influence neurodevelopment, potentially increasing the risk for both TS and immune conditions [67,68].

### 4.4. Physical Health Conditions

Diabetes was significantly more common in the TS group (3%) versus the non-TS group (0.4%) (*p* < 0.001). This higher prevalence is possibly due to Socioeconomic disadvantages, such as reduced access to healthcare and lower physical activity levels, are common among individuals with TS and may contribute to the increased prevalence of diabetes. Additionally, both TS and diabetes might share a common pro-inflammatory profile, characterized by elevated levels of inflammatory cytokines, cortisol, and leptin, which can promote the development of both metabolic disorders and neuropsychiatric conditions [69]. Another crucial factor to consider is the potential impact of medications commonly used to manage TS, particularly antipsychotics. These medications are known to have metabolic side effects, including weight gain, insulin resistance, and increased risk of type 2 diabetes. Long-term use of antipsychotics in individuals with TS may therefore contribute to the higher prevalence of diabetes observed in this population. While these medications are effective in managing the symptoms of TS, their metabolic side effects underscore the importance of careful monitoring and management of metabolic health in individuals receiving these treatments [70].

### 4.5. Limitations

One limitation of this study is that the NSCH dataset does not include information on certain variables such as obsessive-compulsive disorder (OCD), suicidality, and substance use disorders. While this limits the scope of our analysis, the dataset remains a valuable resource for understanding broader trends in neurodevelopmental conditions. Additionally, because the data are parent-reported rather than clinically diagnosed, there is the potential for reporting bias based on parental perceptions.

Another consideration is the dataset’s cross-sectional design, which limits the ability to establish causal relationships. While our findings highlight associations between Tourette syndrome and factors such as low birth weight and prematurity, they do not imply causation. These associations align with prior research on neurodevelopmental conditions, but longitudinal studies are needed to further explore these relationships.

Despite these limitations, the study has several strengths. The NSCH dataset provides a large, nationally representative sample, enhancing the generalizability of our findings. Additionally, by examining multiple socio-demographic and health-related factors, our study contributes valuable insights into the characteristics and potential risk factors associated with Tourette syndrome. Future research could build on these findings by incorporating datasets with more comprehensive clinical variables and employing advanced statistical techniques to further refine our understanding of these associations.

## 5. Conclusions

This study highlights the prevalence and diverse comorbidities of Tourette syndrome in children, with a rate of 0.3% in the U.S. pediatric population. TS is more common in males and adolescents, and is strongly associated with neurodevelopmental, psychiatric, immune-related, and physical health conditions. Comorbidities significantly increase the burden on affected children, impacting their quality of life and overall well-being.

The findings emphasize the importance of early diagnosis and comprehensive, multidisciplinary care to manage both the tics and associated comorbidities. Tailored interventions addressing these coexisting conditions are critical to improving outcomes. Healthcare providers should prioritize routine screening for comorbidities in children with TS to ensure timely intervention and support. The study underscores the need for ongoing research to further understand the complex interactions between TS and its comorbidities, ultimately leading to better management strategies and improved quality of life for affected children.

## Figures and Tables

**Table 1:** Socio-demographic characteristics of study participants.

Socio-Demographic Characteristics	Total (N = 79,236)	With TS (N = 208)	Without TS (N = 79,028)	TS + Prematurity (N = 36)	TS + Low Birth Weight (N = 27)
Mean age (SD) (years)	11.8 ± 4.0	12.7 ± 3.6	10.0 ± 4.5		
Age Groups					
Preschool (3–5 years)	24%	5%	24%	2.8%	3.7%
School (6–10 years)	28%	22%	28%	22.2%	25.9%
Adolescents (11–17 years)	48%	74%	48%	75%	70.3%
Sex					
Male	52%	74%	52%	69.4%	70.4%
Female	48%	26%	48%	30.6%	29.6%
Race					
Hispanic	14%	14%	14%	19.4%	22.2%
White	66%	72%	66%	58.3%	55.6%
Black	7%	6%	7%	8.3%	11.1%
Asian	6%	2%	6%	2.9%	3.7%
Multi-race	7%	4%	7.1%	11.1%	7.4%
Family income relative to FPL					
≥400%	40.3%	39%	40%	36.1%	48.1%
200–399%	30.4%	24%	30%	16.7%	11.1%
100–199%	16.6%	20%	17%	22.2%	11.1%
<100%	13%	16%	13%	25%	29.6%
Highest education of adults					
Less than high school	3%	3%	2.7%	8.3%	7.4%
High school degree	14%	13%	14%	19.4%	14.8%
More than high school	84%	84%	84%	72.2%	77.8%

**Table 2:** Socio-demographic predictors of Tourette syndrome (TS) based on unadjusted and adjusted odds ratios.

Socio-Demographic Characteristics	Unadjusted OR (95% CI)	*p* Value	Adjusted OR (95% CI)	*p* Value
Age Groups				
Preschool (3–5 years)	Reference		Reference	
School (6–10 years)	3.7 (1.9–7.5)	0.000	3.7 (1.9–7.5)	0.000
Adolescents (11–17 years)	7.5 (3.9–14.2)	0.000	7.4 (4.0–14.1)	0.000
Sex				
Male	Reference		Reference	
Female	0.4 (0.3–0.5)	0.000	0.4 (0.3–0.5)	0.000
Race				
Hispanic	Reference		Reference	
White	1.0 (0.7–1.5)	0.889	1.1 (0.7–1.6)	0.700
Black	0.8 (0.4–1.5)	0.494	0.7 (0.4–1.4)	0.364
Asian	0.4 (0.1–1.0)	0.056	0.4 (0.2–1.1)	0.070
Multi-race	0.6 (0.3–1.2)	0.139	0.6 (0.3–1.3)	0.218
Family income relative to FPL				
<100%	Reference		Reference	
100–199%	0.9 (0.6–1.5)	0.881	0.9 (0.6–1.5)	0.732
200–399%	0.6 (0.4–1.0)	0.047	0.6 (0.4–0.9)	0.020
≥400%	0.7 (0.5–1.2)	0.222	0.7 (0.4–1.1)	0.097
Highest education level of adults				
Less than high school	Reference		Reference	
High school degree	0.9 (0.4–2.1)	0.827	1.0 (0.4–2.5)	0.980
More than high school	0.9 (0.4–2.1)	0.905	1.2 (0.5–2.9)	0.597

**Table 3:** Comparison of comorbidities between individuals with and without Tourette syndrome (TS).

Comorbidities	Total (N = 79,236)	With TS (N = 208)	Without TS (N = 79,028)	TS + Prematurity (N = 36)	TS + Low Birth Weight (N = 27)	*p* Value
Neurodevelopmental and Behavioral Disorders						
Attention-deficit hyperactivity disorder	10.3%	49%	10.2%	58.8%	53.8%	0.000
Autism spectrum disorder	3.2%	21%	3.2%	32.3%	40%	0.000
Developmental delay	6%	23%	6%	13.9%	18.5%	0.000
Learning disability	7.6%	28%	7.5%	30.6%	37%	0.000
Behavioral and conduct problems	8%	36%	8%	41.2%	34.6%	0.000
Psychiatric and Neuropsychiatric Disorders						
Depression	5%	25%	5%	33.3%	18.5%	0.000
Anxiety problems	11.4%	60%	11.3%	55.6%	51.8%	0.000
Seizure	0.6%	6%	0.6%	8.3%	7.4%	0.000
Headaches	4%	14.4%	4%	30.5%	14.8%	0.000
Immune-Based Conditions						
Asthma	8%	15.5%	8%	37.1%	30.7%	0.000
Allergies	22%	37%	22%	52.8%	40.7%	0.000
Physical Health Conditions						
Heart disorders	1.4%	2.8%	1.4%	5.6%	7.4%	0.075
Cerebral palsy	0.2%	2.4%	0.2%	5.6%	11.1%	0.000
Diabetes	0.4%	3%	0.4%	5.6%	7.4%	0.000
Down syndrome	0.2%	1.4%	0.2%	5.6%	7.4%	0.000
Hearing problems	1.2%	3.8%	1.2%	13.9%	11.1%	0.001
Genetic disorder	4.6%	14%	4.6%	19.4%	11.1%	0.000
Vision problems	1.5%	4.3%	1.5%	13.9%	11.1%	0.001

## Data Availability

The dataset utilized in this study is publicly available from the Data Resource Center for Child and Adolescent Health website: https://www.childhealthdata.org/learn-about-the-nsch/NSCH (accessed on 1 January 2025).

## Supplementary Materials

The following supporting information can be downloaded at: https://www.mdpi.com/article/10.3390/jcm14051485/s1. Supplementary S1: 2021 National Survey of Children’s Health Questions.
