# Supplementary material for: Unpacking Tourette Syndrome in Children: Insights into Prevalence and Comorbidities from NSCH Data
Source: J Clin Med. 2025 Feb 23;14(5):1485. doi: 10.3390/jcm14051485 (PMC11900335; doi:10.3390/jcm14051485)
Supplement: Supplementary file 1 [file jcm-14-01485-s001.zip › jcm-3447221-supplementary.pdf]

Supplementary S1: 2021 National Survey of Children's Health Questions

Indicator	Description
Health status (ChHlthSt_21)	In general, how would you describe this child's health?
Condition of teeth, age 1-17 years (TeethCond_21)	How would you describe the condition of this child's teeth, age 1-17 years?
Oral health problems, age 1-17 years (OralProb_21)	During the past 12 months, has this child had oral health problems such as toothaches, bleeding gums or decayed teeth or cavities, age 1-17 years?
Breastfed ever, age 0-5 years (BrstEver_21)	Was this child ever breastfed or fed breast milk, age 0-5 years?
Exclusively breastfed, age 6 months-5 years (ExBrstFd_21)	Was this child exclusively breastfed for first 6 months, age 6 months-5 years?
Healthy eating (consumption of sugary drinks, vegetables, fruit), 1-5 years	During the past week, how many times did this child drink sugary drinks/eat vegetables/eat fruit children 1-5 years?
Weight status (BMI) in 4 categories, age 10-17 year (BMI4_21)	What is the weight status of this child based on Body Mass Index (BMI)-for-age, age 10-17 years?
Weight status (BMI) in 3 categories, age 10-17 years (BMI3_21)	Is this child currently overweight or obese, based on Body Mass Index (BMI)-for age, age 10-17 years?
Ever told that child is overweight (ToldOverweight_21)	Has a doctor or other health care provider ever told you that this child is overweight?
Physical activity, age 6-17 years (PhysAct_21)	During the past week, on how many days did this child exercise, play a sport, or participate in physical activity for at least 60 minutes, age 6-17 years?
Time spent outdoors on most weekdays/on an average weekend, age 3-5 years (OutdrsWkDay_21, OutdrsWkend _21)	On most weekdays/on an average weekend, how much time does this child spend playing outdoors, age 3-5 years?
Concerns about weight (WgtConcn_21)	Are you concerned about this child's weight?
Low birth weight (LowBWght_21)	Was this child born with a low birth weight, that is, they weighed less than 2500 grams at birth?
Low or very low birth weight (VeryLBWght_21)	Was this child born with a low birth weight, that is, they weighed less than 2500 grams at birth, or a very low birth weight, that is, less than 1500 grams?

Premature birth (BornPre_21)	Was this child born more than 3 weeks before their due date?
One or more health conditions (Cond2more24_21)	Does this child have current or lifelong health conditions from a list of 24 health conditions?<br>*This is a composite measure of current or lifelong health conditions the child experiences from a list of 24 conditions asked in the survey: allergies (food, drug, insect or other), arthritis, asthma, blood disorders (such as sickle cell disease, thalassemia, or hemophilia), cerebral palsy, cystic fibrosis, diabetes, Down Syndrome, epilepsy or seizure disorder, genetic or inherited condition, heart condition, frequent or severe headaches including migraine (3-17 years), Tourette Syndrome (3-17 years), anxiety problems (3-17 years), depression (3-17 years), behavioral and conduct problem (3-17 years), developmental delay (3-17 years), intellectual disability (3-17 years), speech or other language disorder (3-17 years), learning disability (3-17 years), Autism or Autism Spectrum Disorder (ASD) (3-17 years), Attention Deficit Disorder or Attention-Deficit/Hyperactivity Disorder (ADD or ADHD) (3-17 years), hearing problems, and vision problems.*
Severity of current or lifelong conditions	Would you describe this child's current allergies as mild, moderate or severe?
One or more functional difficulties (Diff2more_21)	*Does this child have one or more functional difficulties from a list of 12 difficulties?*<br>*difficulties asked in the survey. The difficulties are asked about as two different types of questions: (a) During the past 12 months, whether the child had frequent or chronic difficulty with breathing or other respiratory problems; eating or swallowing; digesting food, including stomach/intestinal problems, constipation, or diarrhea; repeated or*

	chronic physical pain, including headaches or other back or body pain; using their hands (0-5 years); coordination and moving around (0-5 years); and (b) whether the child has serious difficulty concentrating, remembering, or making decisions (6-17 years); serious difficulty walking or climbing stairs (6-17 years); difficulty dressing or bathing (6-17 years); difficulty doing errands alone, such as visiting a doctor's office or shopping (12-17 years); deafness or problems with hearing; and blindness or problems with seeing, even when wearing glasses.
Children with special health care needs (CSHCN_21)	Does this child have special health care needs (CSHCN) based on the CSHCN Screener?
Effect of conditions on daily activities (DailyAct_21)	Does this child have health conditions that consistently and often greatly affect their daily activities during the past 12 months?
Bullied others, age 6-17 years? (bully_21)	During the past 12 months, how often did this child bully others, pick on them, or exclude them, age 6-17 years?
Bullied, age 6-17 years (bullied_21)	During the past 12 months, how often was this child bullied, picked on, or excluded by other children, age 6-17 years?
Flourishing for young children, age 6 months-5 years? (flrish0to5_21)	Is this young child flourishing, age 6 months-5 years?
Flourishing for children and adolescents, age 6-17 years (flrish6to17_21)	Is this child or adolescent flourishing, age 6-17 years?
Argues too much, age 6-17 years (argue_21)	How often does this child argue too much, age 6-17 years?
Making and keeping friends, age 6-17 years (MakeFriend_21)	) Compared to other children their age, how much difficulty does this child have making or keeping friends, age 6-17 years?
Prevalence of ADD/ADHD, age 3-17 years (ADHDind_21)	Does this child currently have attention deficit disorder (ADD) or Attention Deficit/Hyperactivity Disorder (ADHD), age 3-17 years?
Severity of ADD/ADHD, age 3-17 years (ADHDSevInd_21)	Would you describe this child's current attention deficit disorder (ADD) or Attention-Deficit/Hyperactivity Disorder

	(ADHD) as mild, moderate or severe, age 3-17 years?
Medication for ADD/ADHD, age 3-17 years (ADHDMed_21)	Is this child currently taking medication for ADD or ADHD, age 3-17 years?
Received behavioral treatment for ADD/ADHD, age 3-17 years (ADHDBehTreat_21)	At any time during the past 12 months, did this child receive behavioral treatment for ADD or ADHD, such as training or an intervention that you or this child received to help with their behavior, age 3-17 years?
Prevalence of autism/ASD, age 3-17 years (AutismInd_21)	Does this child currently have autism or autism spectrum disorder (ASD) including Asperger's disorder, pervasive developmental disorder, age 3-17 years?
Severity of autism/ASD, age 3-17 years? (ASDSevInd_21)	Would you describe this child's current autism or autism spectrum disorder as mild, moderate or severe, age 3-17 years?
Medication for autism/ASD, age 3-17 years (ASDMed_21)	Is this child currently taking medication for autism or autism spectrum disorder, age 3-17 years?
Received behavioral treatment for autism/ASD, age 3-17 years (ASDBehTreat_21)	At any time during the past 12 months, did this child receive behavioral treatment for autism, autism spectrum disorder, Asperger's disorder or pervasive developmental disorder, such as training or an intervention that you or this child received to help with their behavior, age 3-17 years?
Age of diagnosis for autism/ASD, age 3-17 years (ASDAge_21)	How old was this child when a doctor or other health care provider first told you that they had autism, autism spectrum disorder, Asperger's disorder or pervasive developmental disorder?
Type of doctor or health care provider first to tell that child had autism/ASD, age 3-17 years (ASDDrType_21)	What type of doctor or other health care provider was the first to tell you that this child had autism, autism spectrum disorder, Asperger's disorder or pervasive developmental disorder, age 3-17 years?
Medication for ADD/ADHD, autism/ASD, or other emotional/behavioral difficulties, age 3-17 years (MedEmotion_21)	I s this child taking medication for ADD/ADHD, autism/ASD, or difficulties with emotions, concentration, or behavior, age 3-17 years?

Mental, emotional, developmental or behavioral problems, age 3-17 years (MEDB10ScrQ5_21)	Does this child have a mental, emotional, developmental or behavioral (MEDB) problem, age 3-17 years?
Current health insurance status (CurrIns_21)	Is this child currently covered by health insurance or health coverage plans?
Consistency of insurance coverage (InsGap_21)	Did this child have consistent health insurance coverage during the past 12 months?
Type of health insurance (InsType_21)	What type of health insurance coverage, if any, did the child have at the time of the survey?
Adequacy of current insurance (InsAdeq_21)	Is this child's current insurance coverage usually/always adequate to meet their needs?
Adequate and continuous insurance (insurance_21)	Is this child adequately and continuously insured; that is, is their current insurance adequate and were they insured for the entire past 12 months?
Adequacy of insurance coverage for mental health care, age 3-17 years (InsMentH_21)	Thinking specifically about this child's mental or behavioral health needs, how often does this child's health insurance offer benefits or cover services that meet these needs, age 3-17 years?
Out-of-pocket cost for health care (OutOfPckt_21)	including co-pays and amounts from Health Savings Accounts (HSA) and Flexible Spending Accounts (FSA), how much money did you pay for this child's medical, health, dental, and vision care during the past 12 months?
Medical care visit (MedCare_21)	During the past 12 months, did this child see a doctor, nurse, or other health care professional for sick-child care, well-child check-ups, physical exams, hospitalizations or any other kind of medical care (including health care visits done by video or phone)?
Received heath care visits by video or phone (video_21)	During the past 12 months, has this child had any health care visits by video or phone?
Preventive care visit (PrevMed_21)	During the past 12 months, how many times did this child visit a doctor, nurse, or other health care professional to receive a preventive check-up? (A preventive check-

	up is when this child was not sick or injured, such as an annual or sports physical, or well-child visit).
Time with doctor during the preventive check-up (VisitTime_21)	Thinking about the last time you took this child for a preventive check-up, about how long was the doctor or health care provider who examined this child in the room with you?
Doctor spoke with child privately, age 12-17 years (PrivateTalk_21)	At their last medical care visit, did this child have a chance to speak with a doctor or other health care provider privately, without you or another caregiver in the room?
Dental care visit, age 1-17 years (DentCare_21)	During the past 12 months, did this child see a dentist or other oral health care provider for any kind of dental or oral health care, age 1-17 years?
Preventive dental care, 1-17 years (PrevDent_21)	During the past 12 months, did this child see a dentist or other oral health care provider for preventive dental care, such as check-ups, dental cleanings, dental sealants, or fluoride treatments, age 1-17 years?
Received both preventive medical and dental care (MedDentCare_21	) During the past 12 months, did this child receive both preventive medical and dental care?
Received mental health care, age 3-17 years (MentHCare_21)	During the past 12 months, has this child received any treatment or counseling from a mental health professional, age 3-17 years?
Difficulties obtaining mental health care, age 3-17 years (MentHDiff_21)	How difficult was it to get the mental health treatment or counseling that this child needed?
Received care from a specialist doctor (SpecCare_21)	During the past 12 months, did this child see a specialist other than a mental health professional?
Difficulties obtaining specialist care (SpCareDiff_21)	How difficult was it to get the specialist care that this child needed?
Saw an eye doctor (EyeDoctor_21)	Has this child ever (age 0-5 years) or in the past 2 years (age 6-17 years) seen an eye doctor?
Received a vision screening from provider other than eye doctor (VisnScrnOther_21)	Has this child received a vision screening from a provider other than an eye doctor

	ever (age 0-5 years) or during the past 2 years (age 6-17 years)?
Hospital emergency room visit (ERVisit_21)	During the past 12 months, how many times did this child visit a hospital emergency room?
Hospital admission (HospitalStay_21)	During the past 12 months, was this child admitted to the hospital to stay for at least one night?
Alternative health care or treatment (AlterHC_21)	During the past 12 months, did this child use any type of alternative health care or treatment?
Doctor asked about parental concerns, age 0-5 years (DrAskConc_21)	During the past 12 months, did this child's doctors or other health care providers ask if you have concerns about this child's learning, development, or behavior, age 0-5 years?
Developmental screening, age 9 - 35 months (DevScrnng_21)	Did the child receive a developmental screening using a parent-completed screening tool in the past 12 months, age 9-35 months?
Special services for developmental needs (SpecServ_21)	Is this child currently receiving special services to meet their developmental needs such as speech, occupational, or behavioral therapy?
Age started receiving special services for developmental needs (SpSerAge_21	) How old was this child when they began receiving special services?
Medical home (MedHome_21)	Did this child receive coordinated, ongoing, comprehensive care within a medical home?
Personal doctor or nurse (PerDrNs_21)	Do you have one or more persons you think of as this child's personal doctor or nurse
Usual source for sick care (UsualSck_21)	Does this child have a place that they usually go to first when they are sick, or a parent/caregiver needs advice about their health?
Family-centered care (FamCent_21)	If this child received care during the past 12 months, did they receive family centered care?
Difficulties getting referrals, all children (NoRefPrb_21)	During the past 12 months, how difficult was it for this child to get referrals to see any doctors or receive any services?

Effective care coordination (CareCoor_21)	Did this child receive effective care coordination?
Shared decision-making (ShareDec_21)	How often did the child’s family feel partnered in shared decision-making for the child's optimal health?
Transition to adult health care, age 12-17 years (Transition_21)	Did this child receive services needed for transition to adult health care, age 12-17 years?
System of care (SystCare2_21)	Does this child receive care in a well-functioning health system?
Forgone health care (ForgoneCare_21)	During the past 12 months, was there any time when this child needed health care, but it was not received?
Problems paying medical bills (MedBills_21	) During the past 12 months, did your family have problems paying for any of this child’s medical or health care bills?
Frustrated in efforts to get services (frustrated_21)	During the past 12 months, how often were you frustrated in your efforts to get services for this child?
Special education or early intervention plan, age 1-17 years (SpEducPln_21)	Is this child currently receiving services under a special education or early intervention plan, age 1-17 years?
Age started special education or early intervention plan, age 1-17 years (SpEdAge_21)	How old was this child when they began receiving special education or early intervention plan, age 1-17 years?
School engagement, age 6-17 years (SchlEngage_21)	How often does this child engage in school: cares about doing well in school and does required homework, age 6-17 years?
Repeated grade(s) in school, age 6-17 years (ReptGrade_21)	Since starting kindergarten, has this child repeated any grades, age 6-17 years?
Missed school days, age 6-17 years (SchlMiss_21)	During the past 12 months, about how many days did this child miss school because of illness or injury, age 6-17 years?
Participation in organized activities, age 6-17 years (AftSchAct_21)	During the past 12 months, did this child participate in any organized activities or lessons, after school or on weekends, age 6-17 years?
Parent participation in child’s events/activities, age 6-17 years (EventPart_21)	During the past 12 months, how often did you attend events or activities that this child participated in, age 6-17 years?

Participation in community service or volunteer work, age 6-17 years (volunteer_21)	During the past 12 months, did this child participate in any type of community service or volunteer work at school, church, or in the community, age 6-17 years?
Work for pay, age 12-17 years (WorkPay_21)	During the past 12 months, did this child work for pay, including regular jobs as well as babysitting, cutting grass, or other occasional work, age 12 -17 years?
Adult mentor, age 6-17 years (Mentor_21)	Other than you or other adults in your home, is there at least one other adult in this child's school, neighborhood, or community who knows this child well and who they can rely on for advice or guidance, age 6-17 years?
This child's learning, individual items, age 3-5 years	This child's learning – Individual items *Variables in public use data file: K6Q08_R; CONFIDENT; RECOGBEGIN; RECOGABC; RHYMEWORD; CLEAREXP; WRITENAME; COUNTTO; RECSHAPES; COLOR; DISTRACTED; WORKTOFIN; SIMPLEINST; USEPENCIL; PLAYWELL; NEWACTIVITY; HURTSAD; CALMDOWN; TEMPER; MAKEFRIEND; SITSTILL*
This child's learning, individual items, age 1-5 years	This child's learning – Individual items Survey Items Used Survey instrument item number for children 0-5 years: G1(a-k) *Variables in public use data file: ONEWORD; TWOWORDS; THREEWORDS; ASKQUESTION; ASKQUESTION2; TELLSTORY; UNDERSTAND; DIRECTIONS; POINT; DIRECTIONS2; UNDERSTAND2*
Physical health status of mother (MothPhyH_21)	If this child's mother is a primary caregiver and lives in the household, what is the mother's general physical health status?
Physical health status of father (FathPhyH_21)	If this child's father is a primary caregiver and lives in the household, what is the father's general physical health status?
Mental health status of mother (MotherMH_21	) If this child's mother is a primary caregiver and lives in the household, what is the general status of the mother's mental and emotional health?

Mental health status of father (FatherMH_21)	If this child's father is a primary caregiver and lives in the household, what is the general status of the father's mental and emotional health?
Overall health status of mother (MotherHSt_21)	If this child's mother is a primary caregiver and lives in the household, is the mother's physical and mental health both excellent or very good?
Overall health status of father (FatherHSt_21)	If this child's father is a primary caregiver and lives in the household, is their father's physical and mental health both excellent or very good?
Someone living in the household smokes (Smoking_21)	Does anyone living in this child's household use cigarettes, cigars, or pipe tobacco?
Someone smokes inside the home (SmkInside_21)	Does anyone smoke inside this child's home?
Caregiver(s) employment status (EmploymentSt_21)	What is the employment status of caregiver(s) in this child's household?
Children living in "working poor" families (WrkngPoor_21)	Does this child live in a 'working poor' household: that is, a household with income less than 100% of the federal poverty level and at least one caregiver employed full- or part-time?
Family shares ideas, 6-17 years (ShareIdeas_21)	How well can you and this child share ideas or talk about things that really matter, age 6-17 years?
Family reads to children, 0-5 years (readto_21)	During the past week, how many days did you or other family members read to this child, age 0-5 years?
Family sings and tells stories to children, 0-5 years (SingStory_21)	During the past week, how many days did you or other family members tell stories or sing songs to this child, age 0-5 years?
Family eats meals together (MealTogether_21)	During the past week, on how many days did all the family members who live in the household eat a meal together?
Time spent in front of a TV, computer, cellphone or other electronic device (ScreenTime_21)	On most weekdays, about how much time does this child usually spend in front of a TV, computer, cellphone or other electronic device watching programs, playing games, accessing the internet or using social media, not including schoolwork?

Family resilience (FamResilience_21)	Does this child live in a home where the family demonstrates qualities of resilience during difficult times?
Adverse childhood experiences (ACE2more11_21)	Has this child experienced one or more adverse childhood experiences?
Parental aggravation (ParAggrav_21)	Does this child have parents who felt aggravated by parenting during the past month?
Parent receives emotional help with parenting (EmSupport_21)	During the past 12 months, was there someone that you could turn to for day-to day emotional support with parenting or raising children?
Coping with daily demands of raising children (ParCoping_21)	How well do you think you are handling the day-to-day demands of raising children?
Job changes due to problems with child care, age 0-5 years (JobChange_21)	) During the past 12 months, did you or anyone in the family have to quit a job, not take a job, or greatly change your job because of problems with childcare for this child, age 0-5 years?
Left a job, took a leave of absence, or cut back hours due to child's health (StopCutWork_21)	During the past 12 months, have you or other family members left a job, taken a leave of absence, or cut down on the hours you work because of this child's health or health conditions?
Avoided changing jobs to maintain insurance (AvoidChng_21)	During the past 12 months, have you or other family members avoided changing jobs because of concerns about maintaining health insurance for this child?
Time spent providing at home health care for children who needed it (HomeCare_21)	In an average week, how many hours do you or other family members spend providing health care at home for this child?
Time spent coordinating health care for children who needed it (TimeCoord_21)	In an average week, how many hours do you or other family members spend arranging or coordinating health or medical care for this child?
Received childcare from others at least 10 hours/week, age 0-5 years (Care10hrs_21)	Does this child receive care for at least 10 hours per week from someone other than their parent or guardian, age 0-5 years?
Sleep position, 0-12 months (SleepPos_21)	In which position do you most often lay this baby down to sleep now, age 0-12 months?

Child goes to bed same time on weeknights (BedTime_21)	How often does this child go to bed at about the same time on weeknights?
Adequate amount of sleep, age 4 months-17 years (HrsSleep_21)	During the past week, how many hours of sleep did this child get during an average day (count both nighttime sleep and naps) (0-5 years)/on most weeknights (6-17 years), age 4 months-17 years?
Food insufficiency (FoodSit_21)	Which of these statements best describes your household's ability to afford the food you need during the past 12 months?
Received food or cash assistance (FoodCash_21)	Does this child live in a household that received food or cash assistance at any time during the past 12 months, even for month?
Supportive neighborhood (NbhdSupp_21)	Does this child live in a supportive neighborhood?
Safe neighborhood (NbhdSafe_21)	Does this child live in a safe neighborhood?
Safe school, age 6-17 years (SchlSafe_21)	Is this child safe at school, age 6-17 years?
Neighborhood amenities (NbhdAmenities_21)	Does this child live in a neighborhood that contains certain amenities -- parks, recreation centers, sidewalks or libraries?
Presence of detracting neighborhood elements (NbhdDetract_21)	Does this child live in a neighborhood where there is litter or garbage on the street or sidewalk, poorly kept or rundown housing, or vandalism such as broken windows and graffiti?
U.S. children in 3 age groups (age3_21)	What is this child's age? (3 age groups)<br>0-5 years; 6-11 years; 12-17 years
U.S. children in 5 age groups (age5_21)	What is this child's age? (5 age groups)<br>0-3 years; 4-7 years; 8-11 years; 12-14 years; 15-17 years
Sex of child (sex_21)	What is this child's sex?
Race and ethnicity distribution of the child population (race4_21)	What is this child's race/ethnicity? Categories Hispanic; White, non-Hispanic; Black, non-Hispanic; Other, non-Hispanic
Parental nativity (PrntNativity_21)	What is the generational status of this child's parents?
Primary household language (HHLanguage_21)	What is the primary language spoken in this child's home?
Primary household language for Hispanic children (hisplang_21)	What is the primary language spoken in home for Hispanic children?

Family structure of child's household (famstruct5_21)	What is the family structure that this child lives in?
Income level of child's household (povlev4_21)	What is the income level (federal poverty level, FPL) of the household that this child lives in?
Highest education of adult in household (AdultEduc_21)	What is the highest education of adult in this child’s household?
Children who were born in US (BornUSA_21)	Was this child born in the United States?
Number of family members (FamCount_21)	How many of the people living or staying in the child's household are family members?
Military status of adult(s) in the household (MilitarySt_21)	Has this child’s caregiver(s) ever served on active duty in the U.S. Armed Forces, Reserves, or the National Guard?
Received heath care visits by video or phone due to the coronavirus pandemic (VideoCOVID_21)	During the past 12 months, has this child had any health care visits by video or phone because of the coronavirus pandemic?
Child missed, delayed, or skipped preventive check-ups due to the coronavirus pandemic (PrevCOVID_21)	During the past 12 months, did this child miss, delay or skip any preventive check-ups because of the coronavirus pandemic?
Childcare was not available due to the coronavirus pandemic, age 0-5 years (Childcare0to5COVID_21)	During the past 12 months, has this child's regular daycare or other childcare arrangements been closed or unavailable at any time because of the coronavirus pandemic, age 0-5 years?
Childcare was not available due to the coronavirus pandemic, age 6-11 years (Childcare6to11COVID_21)	Long question) During the past 12 months, have any of this child's regular childcare arrangements been closed or unavailable at any time because of the coronavirus pandemic, age 6-11 years? Please include before school care, after school care, and all other forms of childcare that were unavailable.
